# Identification of Potent Acetylcholinesterase Inhibitors as New Candidates for Alzheimer Disease via Virtual Screening, Molecular Docking, Dynamic Simulation, and Molecular Mechanics–Poisson–Boltzmann Surface Area Calculations

**DOI:** 10.3390/molecules29061232

**Published:** 2024-03-10

**Authors:** Hind Yassmine Chennai, Salah Belaidi, Lotfi Bourougaa, Mebarka Ouassaf, Leena Sinha, Abdelouahid Samadi, Samir Chtita

**Affiliations:** 1 Group of Computational and Medicinal Chemistry, LMCE Laboratory, University of Biskra, BP 145, Biskra 707000, Algeria; chennaiyassmine68@gmail.com (H.Y.C.); prof.belaidi@gmail.com (S.B.); lotfi.bourougaa@univ-biskra.dz (L.B.); nouassaf@univ-biskra.dz (M.O.); 2 Department of Physics, University of Lucknow, Lucknow 226010, India; sinhaleena27@gmail.com; 3 Department of Chemistry, College of Science, United Arab Emirates University (UAEU), Al Ain P.O. Box 15551, United Arab Emirates; 4 Laboratory of Analytical and Molecular Chemistry, Faculty of Sciences Ben M’Sik, Hassan II University of Casablanca, Casablanca 7955, Morocco

**Keywords:** Alzheimer’s disease, acetylcholinesterase, HUP, e-pharmacophore, docking, ADMET, molecular dynamics, MM-PBS

## Abstract

Huperzine A (HUP) plays a crucial role in Alzheimer’s therapy by enhancing cognitive function through increased cholinergic activity as a reversible acetylcholinesterase (AChE) inhibitor. Despite some limitations being seen in AChE inhibitors, ongoing research remains dedicated to finding innovative and more effective treatments for Alzheimer’s disease. To achieve the goal of the discovery of potential HUP analogues with improved physicochemical properties, less toxic properties, and high biological activity, many in silico methods were applied. Based on the acetylcholinesterase–ligand complex, an e-pharmacophore model was developed. Subsequently, a virtual screening involving a collection of 1762 natural compounds, sourced from the PubChem database, was performed. This screening yielded 131 compounds that exhibited compatibility with the established pharmacophoric hypothesis. These selected ligands were then subjected to molecular docking within the active site of the 4EY5 receptor. As a result, we identified four compounds that displayed remarkable docking scores and exhibited low free binding energy to the target. These top four compounds, CID_162895946, CID_44461278, CID_44285285, and CID_81108419, were submitted to ADMET prediction and molecular dynamic simulations, yielding encouraging findings in terms of their pharmacokinetic characteristics and stability. Finally, the molecular dynamic simulation, cross-dynamic correlation matrix, free energy landscape, and MM-PBSA calculations demonstrated that two ligands from the selected ligands formed very resilient complexes with the enzyme acetylcholinesterase, with significant binding affinity. Therefore, these two compounds are recommended for further experimental research as possible (AChE) inhibitors.

## 1. Introduction

Alzheimer’s disease (AD) is a neurological illness (i.e., chronic brain damage that causes neuronal death) characterized by a gradual loss of memory and certain intellectual (cognitive) functions that might interfere with everyday activities. AD is the leading cause of dementia, accounting for 60–80% of cases. [1]. This disease manifests itself in multiple cognitive deficiencies, such as memory loss, cognitive disorders, thinking difficulties, and language disorders [1,2]. Current research indicates that increasing brain cholinergic neurotransmission by decreasing the chemical reaction to AChE hydrolysis is the most effective therapy for Alzheimer’s disease, although the pathophysiology of the illness is not entirely understood. Research on acetylcholinesterase (AChE) in chronic Alzheimer’s disease has emerged as a potential area of study. In the central nervous system, AChE is a crucial enzyme that plays an effective role in acetylcholine-mediated neurotransmission. Currently, AChE inhibitors, notably rivastigmine, donepezil, and galantamine, are very common for the treatment of AD. In fact, the reliability of these drugs is weakened by their various side effects, such as hypotension and hepatotoxicity [3]. Despite the side effects of drugs based on AChE inhibition, this research still represents a promising strategy for designing novel medications to treat AD disease [4]. The chemical structure of human acetylcholinesterase (hAChE) comprises a central mixed β-sheet composed of 12 strands surrounded by 14 α-helices.

The integration of e-pharmacophore structure-based methods with molecular docking studies is a highly effective approach for drug discovery. Pharmacophore-based methods enable the identification of critical molecular features that are necessary for a compound’s interaction with a target receptor, enhancing our understanding of the binding requirements. When combined with molecular docking, which simulates the interaction between a small molecule (termed a ligand) and its target receptor, this integrated approach significantly improves the drug discovery process [5].

Our primary aim in this phase was to anticipate the affinity between the interacting amino acid residues of the receptor. This was accomplished by docking the acetylcholinesterase target protein with an Alzheimer’s disease-based ligand, such as the drug Huperzine. This process allowed us to gauge how effectively these ligands bind to the protein acetylcholinesterase, a pivotal element in managing Alzheimer’s disease.

We then characterized the compounds under scrutiny for their ADMET properties, establishing their potential as promising drug candidates that are capable of binding effectively to the acetylcholinesterase protein. These data hold significant promise for the development of new inhibitors that could mitigate and impede the aggression of the hAChE enzyme, which is closely linked to the progression of Alzheimer’s disease.

**Scheme 1:**
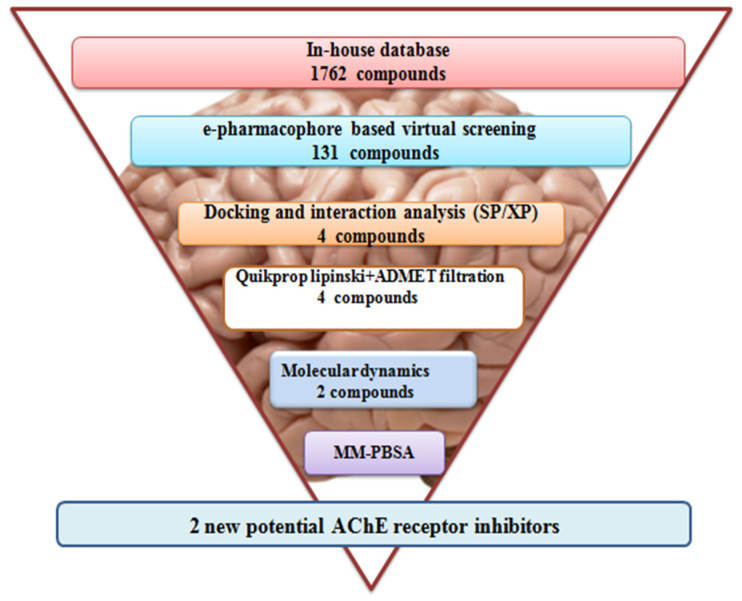
Representation of the methodologies used in the present study.

Furthermore, to evaluate the stability of the generated complexes, we performed molecular dynamics (MDs) simulations and computed various parameters, including the dynamic cross-correlation matrix (DCCM), free energy landscape (FEL), and molecular mechanics–Poisson–Boltzmann surface area (MM-PBSA). These analyses offered a more in-depth understanding of the stability and energetics of the ligand–protein complexes, enriching our comprehension of their interactions and reinforcing their potential as viable drug candidates (Scheme 1).

## 2. Results and Discussion

### 2.1. Development of e-Pharmacophore Hypotheses

**Figure 1:**
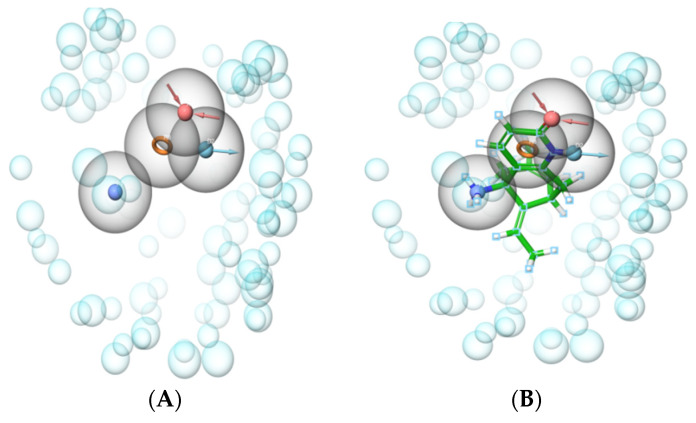
(**A**) Alignment of e-pharmacophore models of acetylcholinesterase. (**B**) Alignment of e-pharmacophore models of acetylcholinesterase with the reference ligand (HUP).

e-Pharmacophore screening methods, renowned for their swiftness, serve as ideal tools for the preliminary screening of extensive molecular libraries. The compounds that are selected after screening are then subjected to a more precise, albeit slower, method, such as Glide SP or other comprehensive techniques, to accurately determine the binding free energy (Figure 1).

**Figure 2:**
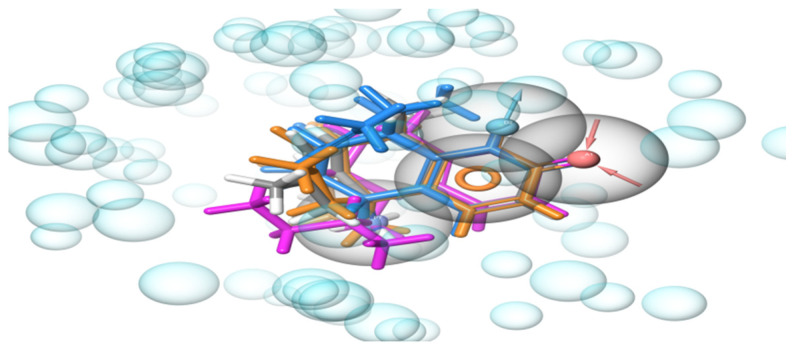
Alignment of respective e-pharmacophore models of 4EY5 with blue color: CID_162895946, gray color: CID_44461278, orange color: CID_44285285, mauve color: CID_81108419.

To gain an in-depth understanding of inhibitors binding to acetylcholinesterase (AChE), we constructed a structure-based pharmacophore model utilizing the acetylcholinesterase–hup complex. By mapping Glide XP energetic terms onto pharmacophore locales, which were determined based on structural and energy data between the protein and the ligand, e-pharmacophore hypotheses were generated from the redocked structure of the HUP ligand in acetylcholinesterase. The e-pharmacophore hypothesis created for the 4EY5 catalytic region consisted of four pharmacophores, highlighted with a 2.00 Å distance, and included one hydrogen bond donor (D2), one hydrogen bond acceptor (A1), one aromatic ring (R8), and one ionizable group (positive ionic) (P7). Figure 2 shows the selected e-pharmacophores for the selected crystal ligand (HUP) and the CID_44461278, CID_162895946, CID_44285285, and CID_81108419 ligands.

### 2.2. Enrichment Calculations

We used an enrichment survey to eliminate pharmacophores lacking meaningful interactions and to focus on pharmacophores for additional virtual screening. Subsequently, we obtained great enrichment and great variety in our hits.

**Figure 3:**
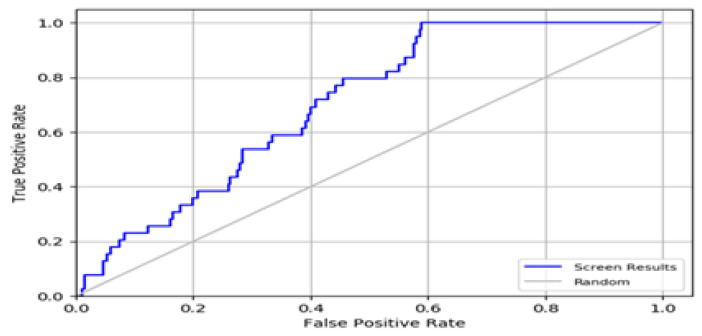
Sketch of the ROC plot used for validation of the e-pharmacophore model.

Multiple metrics, such as the EF, ROC, AUC, and BEDROC curves, were used to validate the model. An EF (1%) score of 2.40 was the greatest rate of active recovery from the dataset in comparison to random selection. Furthermore, the ROC and AUAC values of 0.7 and 0.69, respectively (Table 1), indicate that the model is capable of recovering all the active compounds from the decoy set. The ROC curve is depicted in Figure 3. It is important to note that the model’s BEDROC value was judged to be 0.08, emphasizing its high quality and efficiency in the screening process. Based on the validation results, we believe that this e-pharmacophore model is trustworthy and might be useful for identifying possible hits thoroughly.

### 2.3. Screening e-Pharmacophore-Based Virtual Screening

We embarked on the quest for new compounds with the potential to serve as potent acetylcholinesterase inhibitors, aiming to address the pressing needs of Alzheimer’s disease research. Leveraging data from the PubChem database, we gathered compounds that are analogous to our reference molecule (Huperzine A), underpinned by the principle that structural similarity implies similar properties. Our initial step involved employing a validated pharmacophore model, which robustly confirmed its efficacy. This model served as our filter, sifting through a pool of 1762 compounds. Following a stringent screening based on the feature count and fit values (more than 2.0), we narrowed our selection to 131 compounds. This rigorous computational filtration process represents our foundational aim of identifying promising candidates that could exhibit desirable inhibitory effects on acetylcholinesterase, a critical pursuit in the quest for novel therapeutics targeting Alzheimer’s disease.

### 2.4. Glide Ligand Docking

**Figure 4:**
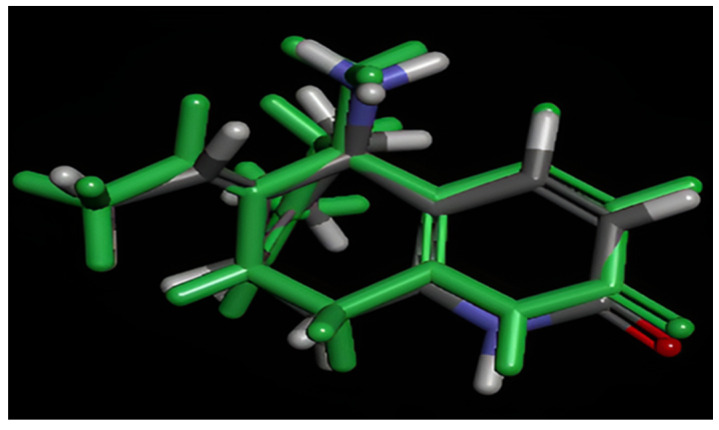
Superimposition image of the X-ray native pose and the redocked pose of the co-crystal ligand (HUP) in the active site of the target PDB-4EY5. Gray: X-ray native pose; green: redocked pose.

After filtering using the pharmacophore model, a pool of 131 potential compounds was identified. Our subsequent step involved platforming a docking study, as described in Section 3.3, with the protein 4EY5 serving as our study’s focal point. To validate our approach, we initially conducted redocking of the co-crystallized ligand at its stated binding site [6]. By comparing the resulting pose with the reported one, as depicted in Figure 4, and computing the root mean square deviation (RMSD) value, we confirmed the fidelity of our docking process. Notably, the RMSD value between the co-crystallized ligand and the redocked ligand was less than 0.1 Å, confirming the accuracy of our docking methodology.

The 131 selected molecules were deemed excessively large for further docking in the higher accuracy mode (XP). Consequently, the initial refinement was carried out through docking in the standard accuracy (SP) mode, narrowing the selection to 40 compounds. The SP mode allows for flexibility by scaling specific potential components, enhancing the adaptability. Conversely, the XP docking approach serves to eliminate false positives, establishing a robust correlation between favorable poses and high ratings.

**Figure 5:**
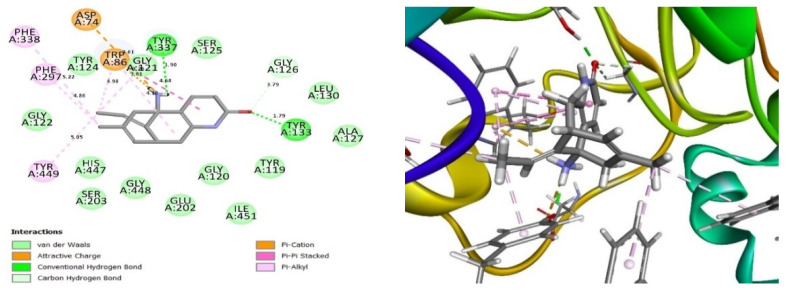
The reference ligand in the active site of the target acetylcholinesterase and its interactions with amino acid residues.

Following this screening, molecular XP docking was performed on the 40 selected compounds using the 4EY5 crystal structure. Subsequently, four compounds exhibiting the most favorable docking scores (−11.436 to −10.680 kcal/mol) and Glide energies (−55.585 to −38.552 kcal/mol) were selected for further investigation, as delineated in Table S1. The ligands were classified based on their docking scores and their interactions with unbound amino acids. The determined interactions were similar to those of the co-crystallized ligands (C15H18N2O, Huperzine A). As illustrated in Figure 5, the co-crystallized Huperzine A (HUP) ligand showed a conventional hydrogen bond with TYR 133 and TYR337; a Pi-Cation and an attractive charge with ASP 74 and TRP 86; a Pi-Alkyl with PHE 338, PHE 297, TYR 449, TRP 86, and TYR 337; and a carbon hydrogen bond with GLY 126 amino acid residue, while the Glide energy value of the given molecule was −46.720 kcal/mol and the docking score was −10.217 kcal/mol (Table S1). The results obtained in the enzyme’s active site, where the ligand binds, and the nature of that interaction are also supported by the literature [6].

**Figure 6:**
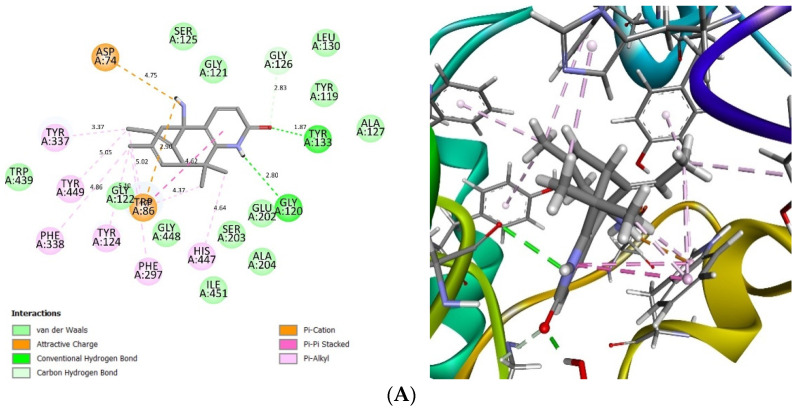

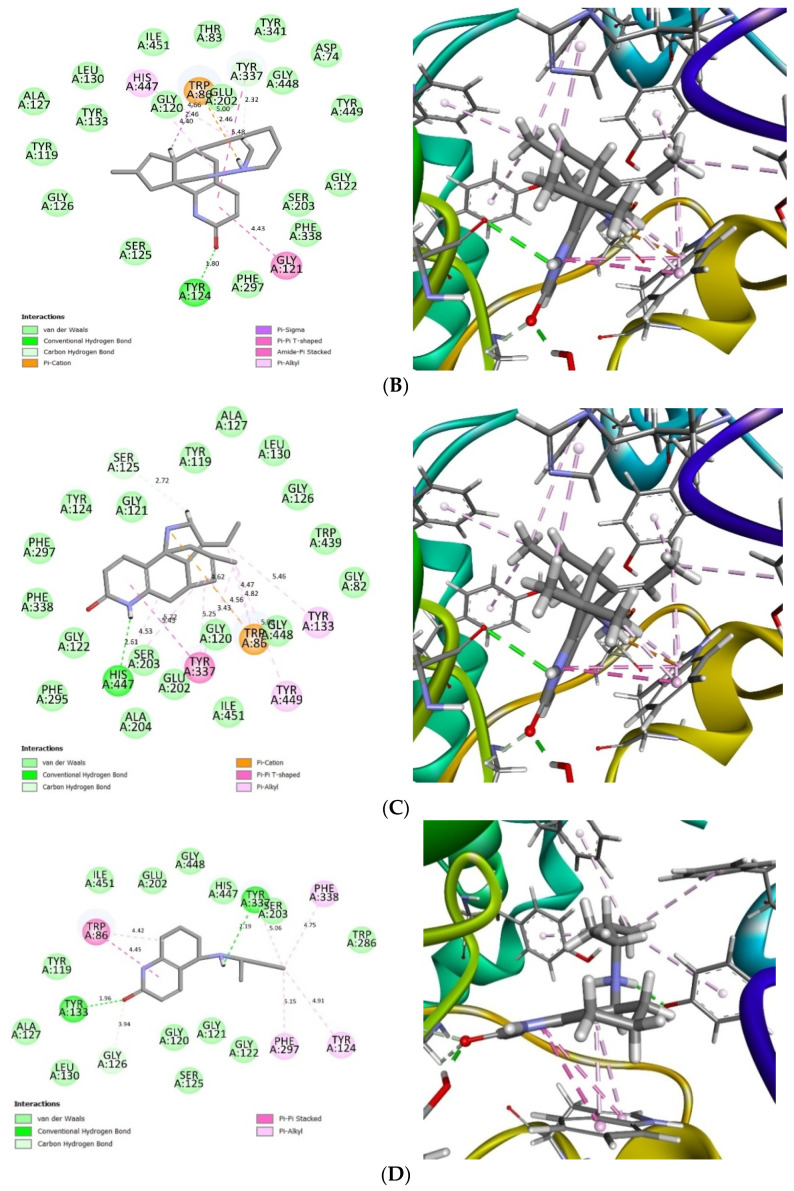
Interactions between amino acid residues and selected molecules (**A**) CID_44461278, (**B**) CID_162895946, (**C**) CID_CID_44285285, and (**D**) CID_81108419 in the active site of the target 4EY5.

The docking scores for the four ligands CID_162895946, 44461278, 44285285, and 81108419 were greater than −10.217 kcal/mol (−11.436, −11.107, −10.792, and −10.680 kcal/mol, respectively). The compounds CID_162895946 and 44461278, which had the highest fitness scores (2.900 and 2.889, respectively), also had the highest docking scores of −11.436 and −11.107 kcal/mol, respectively, with Glide energies of −38.552 and −50.035 kcal/mol, respectively. These lower values indicate that the inhibitor fit into the actual binding site of the target enzyme and that a stable ligand–receptor complex was formed; additionally, both of the top hits interacted with the majority of the key residues that were involved in substrate binding (Figure 6).

As illustrated in Table S2, CID_44461278 showed a strong conventional hydrogen bond, carbon hydrogen bond interactions with TYR 133,GLY 120, and Gly 126 amino acid residues in the active site of the target, and oxygen and nitrogen of the backbone atom; it also showed Pi-Cation interaction and attractive charge with ASP 74 and TRP 86 residues, height Pi-Alkyl interaction with PHE 338, PHE 297, and TYR 449, and one observed interaction of the Pi-stacked type (aromatic interactions).

CID_162895946 exhibited one hydrogen bond and one carbon hydrogen bond interaction with TYR 124 and TYR 337. However, GLY 121 and TYR337 established amide pi-stacks (aromatic interactions) with CID_1628959446. Pi-Alkyl and Pi-Cation interactions were also observed between the proposed ligand and TRP86 and HIS447. Pi-sigma interactions were observed with 86 TRP amino acid residues.

Similarly, the compound CID_44285285 showed an edonehydrogen bond with HIS 447, one carbon hydrogen bond with SER 203, nine Pi-Alkyl interactions with TYR 133, TYR 449, TRP 86, TYR 337, and HIS 447, one Pi-Cation with TRP 86, and one Pi-Pi T-shaped interaction with TYR 337amino acid residues.

On the other hand, the compound CID_81108419 formed two conventional hydrogen bonds with TYR 337 and TYR 133; five Pi-Alkyl bonds with SER203, TYR 124, PHE 338, PHE 297, and TRP 86; one carbon hydrogen bond with GLY 126; and one Pi-Pi stacked bond with TRP86 amino acid residues.

In addition, by comparing the four structures of the proposed hits with the structure of the reference ligand, we found that when carbon chains (closed or open) are added to the structure of the reference ligand, the resulting compounds become more stable, and thus, the drug effectiveness increases. Thus, carbon is an essential element for increasing drug effectiveness. When we changed the basic structure of the aromatic ring of the reference ligand with an open carbon chain (propane) linked to an amine to form an isopropyl amine, we also found that the stability was high, so we can also say that the amine function is essential for increasing the selected biological activity of the compound.

### 2.5. In Silico ADMET Predictions

The four best hits that we found from virtual screening and molecular docking were subjected to ADMET analysis involving the QikProp module in the Maestro and ADMETlab databases. The QikProp tools were used to investigate drug-like behavior by analyzing the absorption, distribution, metabolism, and excretion (ADME), as well as other pharmacokinetic characteristics (Table 2).

In our case, the proposed compounds for Alzheimer’s disease treatment exhibited small molecular masses. Generally, Alzheimer’s disease drugs are typically small in size. This is because these drugs are designed to target specific regions in the brain, aiming to modify chemical processes, enhance cognitive functions, and decelerate the disease progression. Their smaller size allows them to efficiently access crucial brain areas, amplifying their impact and efficacy in mitigating symptoms associated with Alzheimer’s disease.

The observed range of partition coefficients of the lead compounds (QPlogPo/w) values, from 1.521 to 2.596, is promising. This highlights the well-balanced combination of hydrophilic and hydrophobic properties, which is crucial for efficient absorption and distribution within biological systems [7]. This balance indicates optimal permeability, which is essential for the effective uptake and the potential bioavailability of a drug. In the context of Alzheimer’s disease, such favorable partition coefficients are pivotal characteristics for evaluating a compound’s potential effectiveness as a drug candidate. The projected apparent Caco-2 cell permeability factor QPPCaco, measured in nm/s^−1^, exhibited values ranging from 226.689 to 633.027 among these hits. This factor is crucial in estimating cell permeability across biological membranes. Notably, the lead compounds from the PubChem library displayed substantial values across the evaluated criteria, demonstrating drug-like characteristics based on various physicochemical parameters.

In general, the % human oral absorption of the compounds ranged from 80.541 to 92.287, and their water solubility (QPlogS) ranged from −3.199 to −2.033. The pMDCK (cell-permeable parameter) values ranged from 110.034 to 333.880 and were used as a model to evaluate the human intestinal barrier and medication absorption efficiency. The log HERG (K+ channel blockage) values were found to be less than 5. The given QPlogBB ¼ predicted brain–blood partition coefficient penetration numbers show the molecules’ capacity to cross the blood–brain barrier (acceptable range: −3 to 1.2).The predicted brain–blood partition coefficient ranged from −0.055 to 0.444, demonstrating a greater possibility of BBB penetration. The VD (volume distribution) is an important parameter in our research, because the novel chemicals act at the level of the central nervous system; it is estimated in L/kg. The average VD ranged from 0.04 to 20 L/kg; all the compounds had VD values within this required range, indicating that they were properly distributed throughout the body (Table S3). Cytochrome P450s are required for medication safety, persistence, and bioactivation. CYP3A4 is the most significant enzyme in the cytochrome P450 family. More than half of all clinically used drugs interact with it. All the compounds had a positive response to the P450 CYP3A4 substrate. As a result, the target ligands have no problems with metabolism or removal.

According to the QikProp ADMET investigation, every compound meets the standard of five with zero violations (Lipinski’s criteria), and the 0.55 bioavailability score validates the good bioavailability of these compounds. All of the recently discovered acetylcholinesterase inhibitors have synthetic accessibility scores ranging from 3.19 to 4.47, indicating that these compounds are easily synthesized. In order to enhance our understanding of the ADME properties, we utilized the AdmeLab 2.0 software and incorporated the results to support our analysis in Table S3. As shown in Table S3, we observed that all the compounds had a low T 1/2 (half-life time) with a value < 3 h (half-life time), indicating that the presented compounds were easily and rapidly cleared from the human body [8].

All the lead compounds met the pharmacokinetic requirements for a drug-like molecule and were deemed safe for human consumption. The ADMET results are presented in Table 2, Tables S3 and S4. We utilized the web-based tool Protox-II to predict the toxicity of the lead compounds. The computerized prediction of toxicity was based on five different targets associated with adverse medication responses. The hepatotoxicity, carcinogenicity, mutagenicity, cytotoxicity, and LD50 (acute toxicity) of the compounds were evaluated. The four chemicals were found to be nontoxic, with LD50 values greater than 500 mg/kg (Table S4). This research demonstrated that it is possible to boost the biological activity of a natural substance by making slight structural alterations. This study identified the type of alteration that imparts greater AChE inhibitory efficacy to the product, namely, a lipophilic substituent that is capable of facilitating additional hydrophobic interactions with the enzyme. Furthermore, because the predicted log *p* values of the analogs are 0.519 to 2.596, versus 1.494 for Huperzine, the novel analogs may have a better therapeutic profile due to their greater capacity to cross the blood–brain barrier.

### 2.6. MD Simulations

#### 2.6.1. RMSD and RMSF Analysis

**Figure 7:**
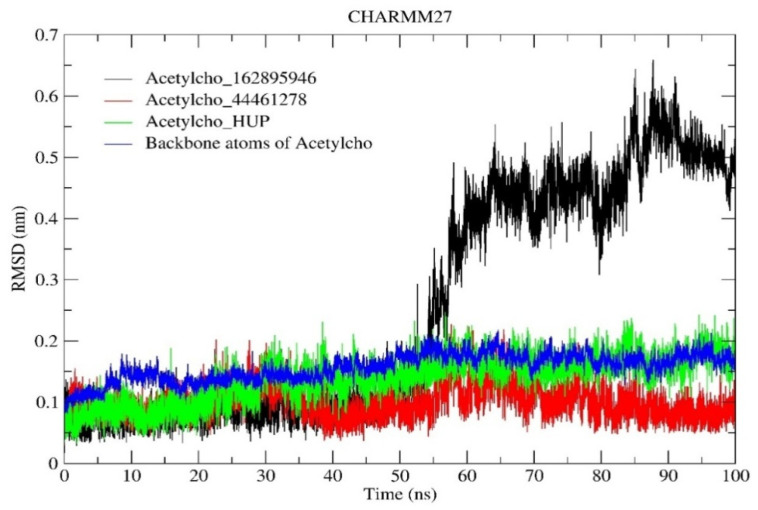
Root mean square deviation (RMSD) analysis of the backbone atoms of acetylcholinesterase and its complexes.

First, we calculated the difference in the structural stability of the three systems’ backbone atoms using the RMSD metric. The RMSD values of the examined complexes were compared to the RMSD profiles of the reference complex (acetylcholinesterase_HUP) and the protein backbone atoms. Figure 7 shows the RMSD profile of each examined system. The RMSD profile of the acetylcholinesterase_44461278 complex was significantly more stable than that of the other complexes after 100 ns of simulation with an RMSD of 0.100 nm than that of the reference complex and protein backbone atoms (with average RMSD values of 0.138 and 0.156 nm, respectively). At the biomolecular level, compound 44461278 formed a highly stable complex with the acetylcholinesterase’s active site, permitting the selectivity to block the vital and biological activities of this protein. Furthermore, the acetylcholinesterase_162895946 complex was comparatively stable during the first 55 ns of the simulation. Following this period, the RMSD values approached 0.5 nm in certain cases until the simulation ended. In addition, the average RMSD value of this complex was 0.138 nm. Finally, the RMSD analysis indicated that the compounds that were identified in this study have the potential to be candidates for Alzheimer’s disease treatment.

**Figure 8:**
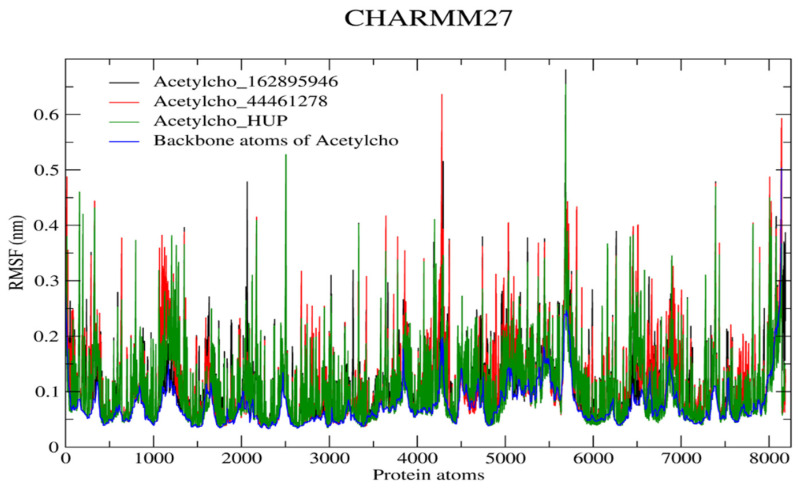
Root mean square fluctuation (RMSF) analysis of the backbone atoms of acetylcholin-esterase and its complexes.

The root mean square fluctuation (RMSF) is a measure of the residual movement of amino acids. More specifically, high RMSF values imply high movement and flexibility in the active site. The extremely low RMSF values characterize a stable and inflexible active site. The RMSF profiles of the three complexes were computed and are shown in Figure 8. The average values of each system were 0.115, 0.120, 0.110, and 0.075 nm for the acetylcholinesterase_162895946, acetylcholinesterase_44461278, and acetylcholinesterase_HUP complex and backbone atom of the protein, respectively. At this point, while binding with the hit compounds compared to the reference complex, the average RMSF values of the amino acid residues were almost identical overall. The RMSF data of each system showed some variations from their initial structures, indicating a dynamic shift. Additionally, all of the fluctuations in the systems matched those of acetylcholinesterase alone, demonstrating the structural and biomolecular stability of the studied systems. Since the RMSF and RMSD analyses yielded similar results, it appears that the proposed compounds may be promising candidates for the development of new and effective drugs for neurological diseases.

#### 2.6.2. Radius of Gyration (Rg) Analysis

**Figure 9:**
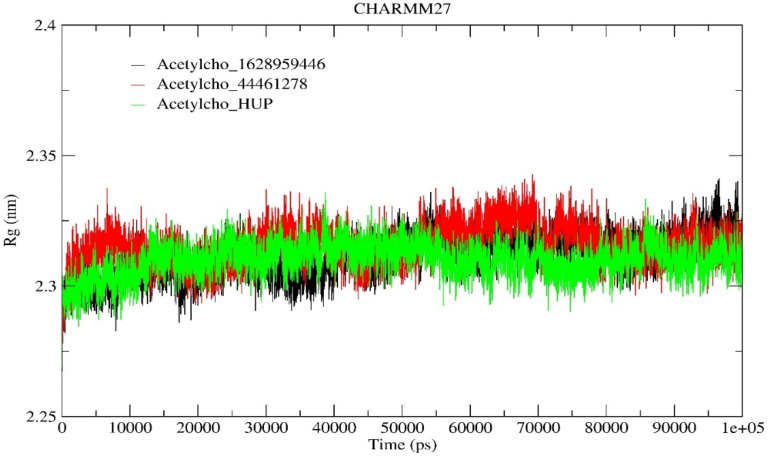
Radius of gyration (Rg) profiles for each system over 100 ns of simulation.

The radius of gyration (Rg) was estimated in this study to reflect the compactness of the three complexes. The calculated average Rg values for the acetylcholinesterase_162895946, 44461278, and HUP complexes were 2.311, 2.315, and 2.309 nm, respectively. There was no structural shift in the acetylcholinesterase configuration in the presence of the hit molecules, and a stable radius of gyration equilibrium was established, implying the stability of the complexes during the 100 ns simulation. The calculated radii of gyration (Rg) of the three systems are shown in Figure 9. This high structural stability will encourage researchers studying neurodegenerative disorders to synthesize and test these compounds in vitro and in vivo.

#### 2.6.3. Solvent-Accessible Surface Area (SASA) Analysis

**Figure 10:**
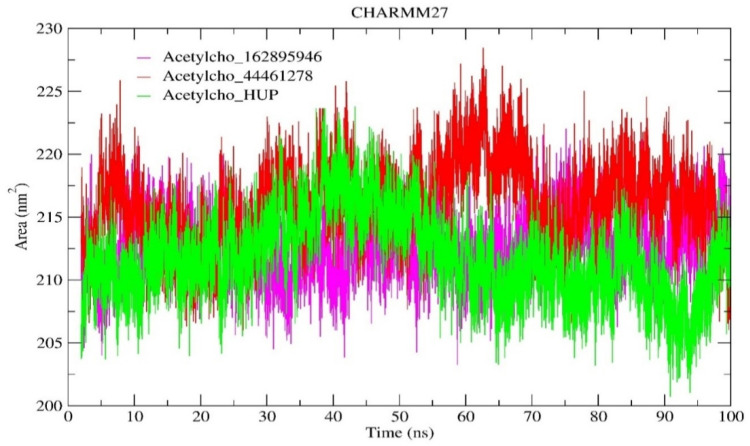
Solvent-accessible surface area (SASA) profiles for each system during 100 ns of simulation.

The solvent-accessible surface area forecasts the dynamical modifications seen throughout the interaction period. As shown in Figure 10, the average SASA value for the reference complex was 211.755 nm^2^, while the average SASA values for both complexes (acetylcholinesterase_162895946 and 44461278) were 212.665 and 216.149 nm^2^, respectively (Table 3). The SASA achieved an equilibrium state without shifting across the simulation, indicating that the acetylcholinesterase configurations were stable in the presence of the two hit compounds. Ultimately, this commonality assists in clarifying the behavior of the hit molecules with the active site of acetylcholinesterase, as well as the occurrence of continuous binding during molecular interactions.

### 2.7. Dynamic Cross-Correlation Matrix (DCCM) Analysis

**Figure 11:**
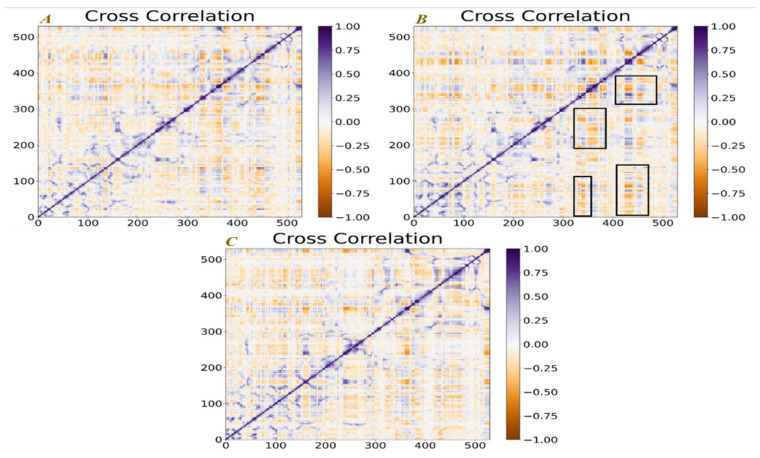
Cα residue cross-correlations computed for the three systems: (**A**) 162895946, (**B**) 44461278, and (**C**) HUP.

The dynamic cross-correlation matrices were used to examine the correlative movements of structural configurations to verify the stability of acetylcholinesterase following interactions with the reference ligand (Huperzine is a clinical drug for Alzheimer disease) and the two hit molecules from the molecular dynamic data. The dynamic cross-correlation maps showed residual shifts in proteins. Figure 11 shows the positive and negative residue movement throughout the 100 ns of simulation. The color measure shows the degree of correlation, with blue representing a positive correlation (+1) and red indicating a negative correlation (−1). A positive correlation reflects that the residues were moving in the same orientation, whereas a negative correlation confirms that the residues were moving in opposite orientations. An inspection of the dynamic cross-correlation matrix maps of the three systems revealed that the correlated motions exhibited by each system were quite comparable. The overall movements that demonstrated an indirect correlation in the acetylcholinesterase_162895946 complex were constant compared to those in the acetylcholinesterase HUP complex, but the movements that indicated a negative correlation increased significantly, especially in the regions denoted by black boxes. Furthermore, at the atomic level, the hit molecule 44461278 formed a stable complex with the active site of acetylcholinesterase. The results of the investigation align with the outcomes of the molecular docking and the measurements obtained from dynamic simulation, confirming the efficacy of the selected compounds in inhibiting the biological functions of acetylcholinesterase.

### 2.8. Free Energy Landscape (FEL)

**Figure 12:**
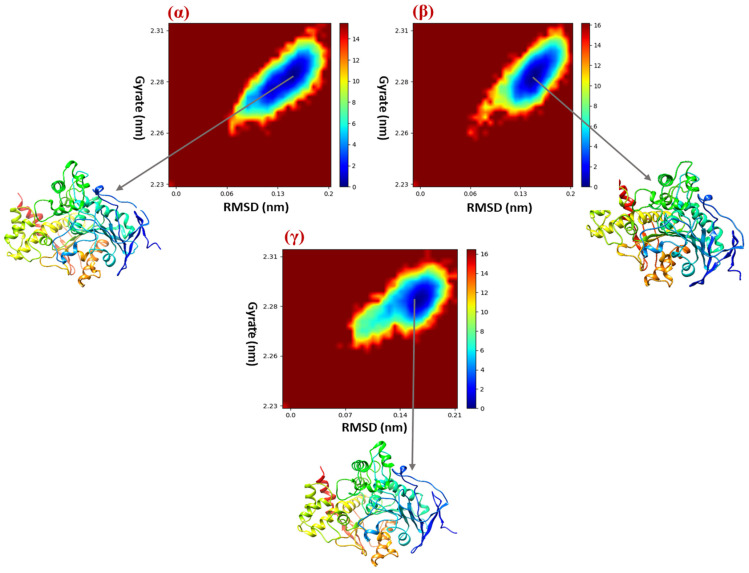
FEL as a function of RMSD (nm) and radius of gyration (nm) for (**α**) acetylcholinesterase_162895946, (**β**) acetylcholinesterase_44461278, and (**γ**) acetylcholinesterase_HUP snapshots of acetylcholinesterase structures from minimum energy basins were extracted.

Demonstrating the nature of structural configurations that are dependent on the free energy landscape is crucial in determining the reason for protein aggregation [9]. To investigate the structural dynamics of acetylcholinesterase after biomolecular interactions with the three compounds, we developed a free energy landscape that represented protein configuration shifts. We selected two reaction parameters for the free energy landscape: the RMSD of acetylcholinesterase (which represents the protein’s structural stability during 100 ns of simulation) and the radius of gyrate (which represents folding). Figure 12 depicts the free energy landscape immediately following the interactions of acetylcholinesterase with the 162895946, 162895946, and HUP molecules. We found a major well located at 0.14 nm and 2.28 nm when interacting with the hit molecule 162895946 and another key well located at 0.16 nm and 2.29 nm when connecting with the hit molecule 44461278. However, upon interaction with a clinical acetylcholinesterase inhibitor (HUP), we clearly observed a centered band at 0.15 nm and 2.28 nm. According to the simulations, the low-energy zones on the free energy maps imply that the entire biomolecular structure and dynamics are reasonably stable. When compound 44461278 was bound to the active site of acetylcholinesterase, the large well from the FEL map confirmed again that this hit molecule forms a highly stable complex with the protein receptor when compared to the drug (HUP) that is currently used to treat Alzheimer’s and neurodegenerative diseases. The extraction of protein structures from the three areas revealed minor conformational changes in the protein structure after interacting with the two hit compounds. The results of the FEL studies were particularly interesting, since they were able to differentiate the structures in terms of biomolecular structure and were similar to the results of the RMSD, RMSF, and DCCM analyses.

### 2.9. Binding Free Energy Calculations

MM-PBSA calculations were executed to conduct a comprehensive examination of the different binding energies that affect the interaction of the three ligands with the acetylcholinesterase receptor. Noncovalent interactions are often prominent during interactions. Hydrophobic energies, hydrogen bonding, electrostatic interactions, and van der Waals energies are among the forces that these energies contribute, negatively or positively, to the total binding energy. The total binding free energy comprises several components, including van der Waals interactions (ΔEVDW), electrostatic interactions (ΔEEEL), polar components (ΔEGB), the nonpolar contribution of the attractive solute solvent interactions to the solvation energy (ΔEDISPER), the nonpolar component of the solvation energy (ΔESURF), the total gas phase molecular mechanic energy (ΔGGAS), and the total solvation energy (ΔGSOLV). Electrostatic interactions were particularly prevalent in the binding of the three complexes (Table 4), with −281.00, −288.37, and −288.42 kJ/mol for acetylcholinesterase_162895946, 44461278, and HUP complexes, respectively. Furthermore, compared to the reference medication HUP, the hit compound 44461278 forms a stable complex with the enzyme receptor. The findings of this investigation showed that these hit molecules may have the ability to inhibit the biomolecular activity of this enzyme, and the molecular docking results were validated.

**Figure 13:**
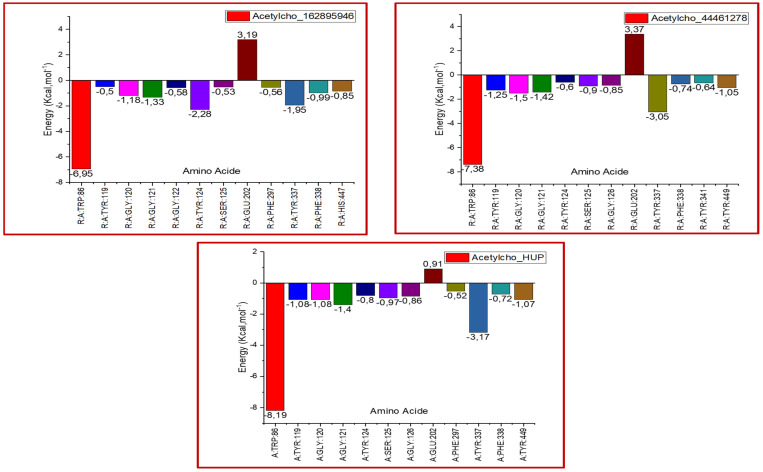
MM-PBSA binding energy decomposition of the three examined systems.

The binding energies of all residues were computed using MM-PBSA calculations and are shown in Figure 13. In addition, the common residues with a binding energy contribution to the three systems were Glu202, Trp86, Tyr119, Gly120, Gly121, Tyr124, Ser125, Glu202, Phe197, and Phe338. In the acetylcholinesterase_162895946 complex, the residues Trp86, Tyr124, Glu202, and Tyr337 contributed −6.95, −2.28, 3.19, and −1.95 kcal.mol^−1^ to the total binding energy, respectively. In the acetylcholinesterase_44461278 complex, the amino acid residues Trp86, Tyr119, Gly121, Glu202, and Tyr449 contributed −6.95, −2.28, 3.19, and −1.95 kcal.mol^−1^ to the total BE, respectively. In addition, in the reference complex with HUP molecules, the amino acid residues Trp86, Tyr119, Gly120, Gly121, and Tyr337 contributed −8.19, −1.08, −1.08, −1.4, and −3.17 kcal/mol to the total binding energy, respectively. All of these data suggest that interactions with amino acids (Trp86, Glu202, and Tyr237) are required to suppress the biochemical and cellular activities of acetylcholinesterase, which might be important in the development of potent treatments for Alzheimer’s disease and other neurodegenerative illnesses.

## 3. Materials and Methods

### 3.1. Dataset

In this work, the data are divided into three parts. The first part contains 39 compounds collected from the literature [10] and is used to validate the in silico e-pharmacophore model. The second Contein-900 molecules were obtained from the Dude Decoys site [11]. These molecules have also been used in the validation of in silico e-pharmacophore models. One thousand seven hundred and sixty-two molecules, which were derived from Huperzine A and retrieved as SDF files from the PubChem database, were employed for screening and docking through in silico e-pharmacophore models.

### 3.2. Preparing the Ligand Structure

Using the PubChem tool https://pubchem.ncbi.nlm.nih.gov/ (accessed on 15 August 2023), 3D ligand structures were downloaded in SDF format and were then exposed to Maestro’s LigPrep panel in Schrödinger Suite 2018 (https://www.schrodinger.com/products/ligprep (accessed on 15 August 2023)). The resulting energy-minimized conformers, chiralities, and ionized states at pH 7.0 ± 2.0 were preserved, and salts were removed using Epik (4.1); furthermore, LigPrep ensured the exclusion of structurally unwanted ligands. The force field was constructed to minimize the number of prepared ligands using optimization potential liquid simulation (OPLS-3). This force field was developed specifically for simulating small molecules.

### 3.3. Preparing the Protein Structure

The X-ray crystal structure of acetylcholinesterase (hAChE), which forms a stable complex with the inhibitor, accession code Huperzine A (HUP, PDB ID: 4EY5) [12], was retrieved from the RCSB Protein Database. The 3D crystal structure of the enzyme was preprocessed using the Protein Preparation Wizard (Maestro) program (Schrodinger Suite 2018 Protein Preparation Wizard; Epik Schrödinger, LLC: New York, NY, USA, 2018). This step is crucial for ensuring that the protein is properly prepared for molecular docking investigations, so that it can correct any problems in the protein, for example, by removing missing atoms, rings, or side chains, as well as unwanted components, and correcting the bonding sequence [2].

### 3.4. Receptor Grid Generation

Grid boxes can usually be created using the module “receptor grid generation” [13]. The workspace ligand center of mass has a closed box to represent the activity of the receptor. The process described in the “receptor grid generation” module was used to determine the centers of the bound ligands in the target, resulting in the creation of grid arrays of Maestro Glide v8. 3, Schrödinger LLC, New York, NY, USA, 2018-2.

### 3.5. Development of the e-Pharmacophore Hypothesis

The ensemble of steric and electronic properties that are required to ensure ideal supramolecular interactions with a particular pharmacological target structure and to initiate (or inhibit) its biological response is referred to as a “pharmacophore”. The phase module of the Schrödinger software, LLC, New York, NY, USA, 2018was used to exploit and develop e-pharmacophore functions using XP descriptor data [14]. The hydrogen bond (HB) donor (HBD), the HB acceptor (HBA), the hydrophobic group (H), the positively and negatively ionizable groups (P) and (N), respectively, and the aromatic ring (R) were used to generate e-pharmacophore sites [15]. These chemical attributes were ranked using the Glide XP estimated energy contribution to anchor the ligand within the binding pocket. [16] Following that, the created library was screened using the Phase Ligand Screening panel against the generated pharmacophoric characteristics, yielding a collection of compounds that matched the features.

### 3.6. Enrichment Calculations

The hypothesis was further tested for robustness and the capacity to discriminate between active and inactive substances using enrichment analysis. For this reason, the enrichment calculator program included in the Schrodinger suite was used.

The decoy collection, which included 900 chemicals, was obtained from the DUD: Directory of Useful Decoys database [11]. The model’s integrity was subsequently tested using the DUD dataset and 39 known acetylcholinesterase inhibitors. Eventually, the query model picked and evaluated the dataset, yielding an array of statistical parameters. The receiver operating characteristic (ROC) curve, enrichment factor (EFROC), area under the accumulation curve (AUAC), and BEDROC were used to verify the e-pharmacophore model using a prepared dataset concatenation [Equation (1)], where the enrichment factor (EF) is described as the proportion of drugs that are already defined when part of a database is analyzed [17], and the F (X%) is the proportion of known agents returned after screening X% of the database; we initially focused on EF (1%) [18]. Another enrichment metric, Boltzmann-enhanced receiver operating characteristic discrimination (BEDROC), was used to guarantee that the pharmacophore results were meaningful [19]. To compare these values, we utilized α = 8.0, α = 20.0, and α = 160.9. It was determined that selecting α = 20.0 was an acceptable number for virtual screening.

EF = (Ha × D)/(Ht × A)(1)

### 3.7. Glide Ligand Docking

The suggested compounds were Glide-docked using previously produced receptor lattices and ligand molecules. To determine the best possible interactions between drugs and receptors, the Glide ligand docking program was used. Docking calculations were performed in SP (standard precision) and XP (ultra-precision) [5,18,20], using the OPLS 2005 force field [16]. The docking methodology was carried out in flexible docking mode, which automatically produces conformations for every input ligand. A sequence of hierarchical filters was applied to the obtained ligand positions to assess the ligand–receptor interactions. Using a grid-based method based on modeling of the empirical ChemScore function [21], the primary filter assesses the complementarity of the ligand–receptor interaction and the spatial fit of the ligand to the designated active site. The algorithm detects favorable hydrophobic interactions, hydrogen bonding, and metal linkage interactions and penalizes steric conflicts. Poses that meet these basic criteria go on to the last step of the algorithm, where the lattice-approximated OPLS interaction energy between the unbound ligand and receptor is assessed and minimized. Finally, the Glide score was used to rescore the minimization posture. The XP–Glide scores of the active compounds were consolidated, and the fitness scores of each ligand were compared. The docking results were analyzed using Biovia Discovery Studio. 4.5.12 http://www.3dsbiovia.com (accessed on 10 October 2023).

### 3.8. Structure-Based Virtual Screening

Virtual screening is a powerful method that is used to find active ingredients or lead molecules and has been incorporated into the drug discovery process of several pharmaceutical companies [22]. These active site screening studies were performed using the Schrödinger Suite, e-pharmacophore, and docking virtual screening workflows. For the electronic pharmacophore approach, it is necessary to identify the most favorable sites for the distribution of energy (greater than 1.0 kcal mol^−1^). When screening molecules, at least four sites must match the hypothesis. The distance adjustment tolerance was set at 2.0 in order to achieve a harmonious equilibrium between tight and loose alignment. The database hits were arranged in order of their fitness score, which is a measure of how well the aligned ligand conformers match the hypothesis. This evaluation considers factors such as the RMSD position agreement, vector alignment, and volume parameters. The fitness score function is an equally weighted combination of these three terms, ranging from 0 to 3, as implemented in the standard database filter in the phase, version 3.3, Schrödinger, LLC, New York, NY, USA, 2018. After pharmacophore-based screening, as indicated by the fitness score, the 131 best hitswere chosen for molecular docking investigation.

### 3.9. In Silico ADMET Prediction

After identifying the best hits via pharmacophore-based virtual screening and molecular docking investigations, we were interested in identifying the ADMET (absorption (A), distribution (D), metabolism (M), excretion (E), and toxicity (T)) properties of the identified compounds. We next investigated whether these compounds exhibited drug-like properties using another tool called QikProp [23]. The QikProp module version 5.4 of Maestro, Schrödinger was used to calculate molecular descriptors (excipients, physicochemical, biochemical, pharmacokinetic, and deleterious properties) and predict the ADMET profiles of these compounds. The SwissADME data site was used to calculate the bioavailability score and the synthetic accessibility values (http://www.swissadme.ch) (accessed on 7 December 2023). Toxicity refers to the degree to which a substance causes damage to an organism or its underlying structures. Compound toxicity prediction is important for reducing the cost and effort required for preclinical and clinical studies of drugs [24]. Toxicity assessment was also performed using the ProTox platform (https://tox-new.charite.de) (accessed on 8 December 2023) and the ADMETlab2.0 server (https://Pharmaceutical, accessed on 9 December 2023, Fronts Vol. 4 No. 4/2022 © 2022). The anticipated harmfulness, cytotoxicity, mutagenicity, and cancer-causing features of the chosen compounds were determined.

### 3.10. Molecular Dynamic Simulation (MDS)

The biomolecular stability of the generated complexes was assessed through molecular dynamic simulations for 100 ns using Gromacs-2023 on a GPU system. In this phase, the two best-hit molecules (CID_162895946 and CID_44461278) were chosen due to their good binding affinities to human acetylcholinesterase and their pharmacokinetic features. We subsequently simulated the complex acetylcholinesterase Huperzine A as a reference complex, considering the same parameters. While the manuscript maintains consistency by employing the CHARMM27 force field across all systems, it is important to note that CHARMM27 was recently incorporated into the GROMACS package and remains operational from version 4.5 onwards. Moreover, studies have suggested that the CHARMM27 force field performs favorably in protein characterization compared to other force fields [25]. However, despite its continued availability and demonstrated utility, it is crucial to acknowledge that CHARMM27 is considered outdated in the context of current force field development. The choice of CHARMM27, while understandable for consistency purposes, may still introduce limitations to the study’s findings due to potential discrepancies with more recent force fields. Notably, CHARMM27 parameters may not accurately capture certain molecular interactions or structural features compared to more contemporary force fields. Therefore, it is essential to consider the potential impact of these limitations on the interpretation of the results. Future studies may benefit from evaluating alternative force fields that better reflect contemporary insights into molecular behavior. Despite these considerations, the findings presented in this manuscript offer valuable insights, albeit within the context of the chosen force field’s limitations. The Swiss Param platform was employed to create the topology data of the selected hit molecules [26]. In addition, the acetylcholinesterase topology file was created using the CHARMM27 all-atom force field [27,28]. After generating topology files, each system was immersed (solvated) in a cubic box using the transferable intermolecular potential with three point (TIP3P) water model. Additionally, Na+ and Cl− ions were introduced to balance the overall charge. The energy of each neutralized system was reduced to a minimum by using the steepest descent and conjugate gradient methods until the maximum force exerted on the system was less than 10.0 kJ/mol. The v-rescale approach was used to couple each system at a temperature of 300 K for 100 ps, with a coupling value of 0.1 ps, during the NVT equilibration phase. Subsequently, the NPT equilibration phase was carried out for 100 ps, utilizing a Berenson pressure-coupling system with a coupling constant of 2.0 ps. [29,30].

To understand the biomolecular stability and dynamics of the hit molecules within the human acetylcholinesterase active site and their effects on vital and biological functions, the main parameters were evaluated. The root mean square deviation (RMSD) was employed to determine the changes in the backbone atoms of human acetylcholinesterase. The root mean square fluctuations (RMSFs) were used to quantify the relative variations in the levels of each amino acid. The radius of gyration (Rg) was used to figure out the overall compactness. To assess the overall stability of the generated complexes, we calculated the solvent-accessible surface area (SASA).

### 3.11. Dynamic Cross-Correlation Matrix (DCCM)

In order to analyze the collective movements of the studied systems, the gmx covar tool was used to generate the dynamic cross-correlation matrix (DCCM), which represents the shifts of protein Cα atoms during dynamic time [31]. A covariance matrix measuring the correlation aspect of atomic shifts was constructed between atoms i and j. The cross-correlation can be calculated using the following equation [32]:

(2) $${DCCM}_{\left(i,j\right)}=\frac{\langle \Delta riX\Delta rj\rangle }{\sqrt{\langle {\Delta ri}^{2}\rangle }\sqrt{\langle {\Delta rj}^{2}\rangle }}$$

where Δ*ri* and Δ*rj* refer to molecular movement vectors representing atoms *i* and *j* from their average location, regardless of the period interval.

### 3.12. Free Energy Landscape (FEL)

Using the conformation sampling technique, the protein’s native structural configuration was determined. For the purpose of generating the free energy landscape, we employed two critical components as reaction coordinates in our investigation: structural deviation and protein gyration. The energy landscape was computed with the two aforementioned components, applying the following equation [33]:

ΔG (p1, p2) = −k_B_ T ln*ρ*(p1, p2) (3)

where k_B_ is the Boltzmann constant, ΔG represents the Gibbs free energy of state, and T signifies the simulation temperature. The two-dimensional free energy landscapes were produced using the system’s joint distributions of probabilities, ρ(p1, p2), using two separate response coordinates, p1 and p2.

### 3.13. Binding Free Energy Calculations

One of the most prevalent methods for determining the binding free energy of a protein–ligand complex is the molecular mechanics–Poisson–Boltzmann surface area (MM-PBSA) [34]. The approach defined in the g_mmpbsa tool was employed in order to conduct the binding free energy (ΔGbind) assessment for the MM-PBSA [35]. The binding free energy was calculated in the following manner:

ΔG_bind_ = G_complex_ − (G_protein_ + G_ligand_) (4)

where G_complex_ represents the binding energy of the native protein, and G_protein_ and G_ligand_ are the binding energies of the protein and ligand, respectively.

## 4. Conclusions

In this work, an e-pharmacophore hypothesis strategy was used to identify potent AChE inhibitors from the PubChem database. The e-pharmacophore model design is composed of a hydrogen bond donor (D2), a hydrogen bond acceptor (A1), an aromatic ring (R8), and an ionizable (positive ionic) group (P7). The model’s quality was assessed using rigorous enrichment analysis. The bioactive compounds were sorted based on their scores during the phase selection; the information collected from their alignment was used to identify several novel HUP-substituted compounds that may act as AChE inhibitors. The developed molecules were also docked in SP and XP modes, and certain derivatives, such as CID_44461278, CID_1628959446, CID_44 285285, and CID_81108419, exhibited high docking scores compared to those of the co-crystallized ligand. The pharmacokinetic features of these four top hits were subsequently investigated using ADMET analysis. Notably, for all the selected compounds, the pharmacokinetic properties were within the required range. In addition, the biomolecular stability and dynamics of the generated complexes demonstrated the pharmacological potential of the developed inhibitors for blocking the enzymatic and biological activities of AChE. Overall, these results are incredibly significant and might lead researchers to develop novel AChE receptor inhibitors for the treatment of Alzheimer’s disease.

## Figures and Tables

**Table 1:** Validation of the e-pharmacophore model.

Metrics	EF (1%)	BEDROC	ROC	AUAC
Values	2.40	0.08	0.70	0.69

**Table 2:** Estimated physicochemical and pharmacokinetic parameters, bioavailability score, and synthetic accessibility properties of QikProp and SwissADME.

Hits	MW g/mol	Lipinsky	QPlogPo/w	QPlogS	QPlogHERGd	QPPCaco	QPPMDCK	% of Human Oral Absorption	QPlogBB	Bioavailability Score	Synthetic Accessibility
CID_162895946	270.374	0	2.596	−2.958	−4.438	633.027	333.880	92.287	0.444	0.55	4.46
CID_44461278	270.374	0	2.079	−2.934	−4.734	226.689	110.034	81.277	−0.054	0.55	4.47
CID_44285285	268.35	0	2.265	−3.199	−4.952	246.804	120.624	83.028	−0.046	0.55	4.46
CID_81108419	220.31	0	1.521	−2.033	−4.709	313.894	156.427	80.541	−0.055	0.55	3.19
Huperzine, A	242.320	0	1.494	−2.332	−4.706	175.952	83.674	75.880	−0.142	0.55	4.30

MW is the molecular weight; QPlogPo/w is the octanol/water partition coefficient; QPlogS is the projected aqueous solubility; and log S: S in moles/l is the concentration of the solute in a saturated solution that is in equilibrium with the crystalline solid. QPPCaco is the projected apparent Caco-2 cell permeability in nm/s; Caco-2 cells are a model for the gut–blood barrier. Recommended values: QPPCaCo = <25 POOR, >500 GREAT. QPlogHEGd: predicted inhibitory concentration (IC50) for blocking HERG K+ channels. QPlogHEGd is was expected to be less than −5. The apparent MDCK cell permeability was predicted in nm/sec (<25 poor, >500 great). QPlogBB is the expected brain–blood partition coefficient; percent human oral absorption = <20 POOR, >80 great; QPlogBB = −3.0 to 1.2; Recommended settings as per the criteria provided in Schrodinger’s Maestro software version 3.3, LLC, New York, NY, USA, 2018 package documentation.

**Table 3:** The average values of different parameters obtained through MD simulation.

Parameters	Acetylcholinesterase_162895946	Acetylcholinesterase_44461278	Acetylcholinesterase HUP
RMSD (nm)	0.254	0.100	0.138
RMSF (nm)	0.115	0.120	0.110
Rg (nm)	2.311	2.315	2.309
SASA (nm^2^)	212.665	216.149	211.755

**Table 4:** The binding free energy in kcal.mol^−1^ for the three studied systems, determined via MM-PBSA calculations.

	Acetylcholinesterase_162895946	Acetylcholinesterase_44461278	Acetylcholinesterase HUP
ΔE_VDW_	−38.67	−36.79	−35.42
ΔE_EEL_	−281.00	−288.37	−288.42
ΔE_GB_	286.30	288.64	290.01
ΔE_SURF_	−4.48	−4.51	−4.31
ΔG_GAS_	−319.66	−325.15	−323.85
ΔG_SOLV_	281.82	284.13	285.70
Δ_TOTAL_	−37.85	−41.02	−38.15

## Data Availability

Data are contained within the article and Supplementary Materials.

## Supplementary Materials

The following supporting information can be downloaded at https://www.mdpi.com/article/10.3390/molecules29061232/s1, Table S1: Structure, molecular formula, docking score (SP, XP) kcal/mol, glide energy and fitness score of the 4 selected compounds and reference ligand; Table S2: Representations of the molecular interactions between the CID_44461278, CID_162895946, CID_44285285 and CID_81108419 ligands and the acetylcholinesterase receptor; Table S3: ADMET properties of reference compound and selected hits using AdmeLab; Table S4: Toxicity prediction of the reference compound and selected hits using Protox-II.
