# Supplementary material for: Identification of Potent Acetylcholinesterase Inhibitors as New Candidates for Alzheimer Disease via Virtual Screening, Molecular Docking, Dynamic Simulation, and Molecular Mechanics–Poisson–Boltzmann Surface Area Calculations
Source: Molecules. 2024 Mar 10;29(6):1232. doi: 10.3390/molecules29061232 (PMC10974877; doi:10.3390/molecules29061232)
Supplement: Supplementary file 1 [file molecules-29-01232-s001.zip › molecules-2879349-supplementary.pdf]

Supplementary Material

Table S1. Structure, molecular formula, docking score (SP, XP) kcal/mol, glide energy and fitness score of the 4 selected compounds and reference ligand

Hits	Compound structure	Molecular formula	Docking score (SP) kcal/mol	Docking score (XP) kcal/mol	Glide energy kcal/mol	Fitness score
CID_162895946	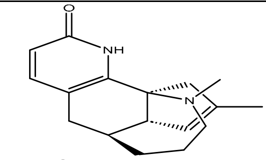	C17H22N2O	-11.387	-11.436	-38.552	2.900
CID_44461278	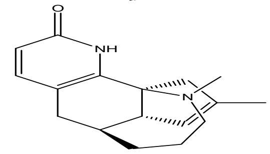	C17H22N2O	-11.026	-11.107	-50.035	2.889
CID_44285285	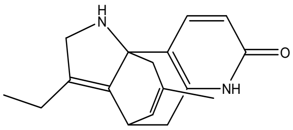	C17H20N2O	-10.724	-10.792	-40.765	2.754
CID_81108419	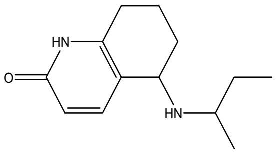	C13H20N2O	-10.672	-10.680	-55.585	2.244
Huperzine A	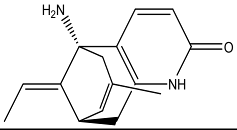	C15H19N2O	-10.120	-10.217	-46.721	***

Table S2. Representations of the molecular interactions between the CID_ 44461278, CID_162895946, CID_44285285 and CID_81108419 ligands and the acetylcholinesterase receptor

**Compound**	**Residues**	**Interaction type**
**CID_ 44461278**	TYR 133, GLY120	ConventionalHydrogen Bond
	GLY 126	Carbone Hydrogen Bond interactions
	TRP 86	Pi-Cation interaction
	ASP 74	Attractive Charge
	HIS 447 PHE 297 TRP 86 TYR124 PHE338 TYR 449 TYR 337	Pi-Alkyl interaction
	TRP 86	Pi-stacked type
**CID_162895946**	TYR124	ConventionalHydrogen Bond
	TYR 337, GLY 121	Amide Pi-stacked
	HIS447, TRP 86	Pi-Alkyl interaction
	TRP 86	Pi-Cation
	TYR 337	CarbonHydrogen Bond interactions
	TRP 86	Pi-Sigma
**CID_44285285**	HIS 447	ConventionalHydrogen Bond
	SER 125	Carbon Hydrogen Bond interactions

	133, TYR 449, TRP 86, TYR 337,HIS 447	Pi-Alkyl interaction
	TYR 337	Pi-Pi T-shaped
	TRP 86	Pi-Cation
**CID_81108419**	TYR 337, TYR 133	ConventionalHydrogen Bond
	SER203, TYR 124, PHE 338, PHE 297, TRP 86	Pi-Alkyl interaction
	TRP 86	Pi-Pistacked

Table S3. ADMET properties of reference compound and selected hits using AdmeLab

Hits	VD (Volume Distribution)	P450 CYP3A4 substrate	T 1/2 (Half Life Time)
**CID_162895946**	0.891 L/kg	++	1.7 h
**CID_44461278**	0.486 L/kg	+	1.8 h
**CID_44285285**	0.812 L/kg	+	1.7h
**CID_81108419**	0.772 L/kg	+	1.3 h
**Reflig**	0.424 L/kg	+	1.6 h

Table S4. Toxicity prediction of the reference compound and selected hits using Protox-II

Hits	Hepatotoxicity	Carcinogenicity	Mutagenicity	Cytotoxicity	LD50 (LD50 of acute toxicity)
CID_1628959446	Inactive	Inactive	Inactive	Inactive	528.406 mg/kg
CID_44461278	Inactive	Inactive	Inactive	Inactive	550.767 mg/kg
CID_44285285	Inactive	Inactive	Inactive	Inactive	524.467 mg/kg
CID_81108419	Inactive	Inactive	Inactive	Inactive	948.519 mg/kg
Huperzine A	Inactive	Inactive	Inactive	Inactive	225.109 mg/kg
